# Alternative Crops for the European Tobacco Industry: A Systematic Review

**DOI:** 10.3390/plants13020236

**Published:** 2024-01-15

**Authors:** Antonios Mavroeidis, Panteleimon Stavropoulos, George Papadopoulos, Aikaterini Tsela, Ioannis Roussis, Ioanna Kakabouki

**Affiliations:** Laboratory of Agronomy, Department of Crop Science, Agricultural University of Athens, 118 55 Athens, Greece; antoniosmauroeidis@gmail.com (A.M.); stavropoulosp@aua.gr (P.S.); gpapadopoulos@aua.gr (G.P.); aikaterinitsela@gmail.com (A.T.); iroussis01@gmail.com (I.R.)

**Keywords:** alternative crops, *Nicotiana tabacum*, smoking products, tobacco alternatives

## Abstract

Tobacco (*Nicotiana tabacum* L.) is a major industrial crop that has being cultivated for centuries for the manufacturing of cigarettes, cigars, and other smoking products. Due to its negative effects on both human health and the environment, the European Union has adopted strict policies that aspire to reduce the consumption of tobacco. Herbal cigarettes are alternative smoking products that are often advertised as healthier than conventional tobacco cigarettes and are especially popular in Asian markets. Even though the available literature suggests that they are equally detrimental to human health, the introduction of tobacco-alternative crops (TACs) to the European tobacco industry could smoothen the abandonment of tobacco, and eventually smoking products altogether, in the EU. The aim of the present systematic review was to compile a list of possible TACs that could be incorporated in the European smoking industry, and highlight their strengths and weaknesses. The most dominant crops in the literature (and in the existing market products) were calendula (*Calendula officinalis* L.), mullein (*Verbascum thapsus* L.), ginseng (*Panax ginseng* C.A.Mey.), tea (*Camellia sinensis* (L.) Kuntze), chamomile (*Matricaria chamomilla* L.), and mentha (*Mentha* spp.). Even though these crops are promising, further research is required for their incorporation in the European tobacco industry.

## 1. Introduction

*Nicotiana tabacum* L., or simply tobacco, is one of the most successful industrial crops worldwide [1]. Tobacco is primarily cultivated for its leaves, which are used for the production of cigarettes and other smoking commodities [2]. It has been estimated that as of 2020, more than 1 billion people around the globe consume tobacco products [3]. In the European Union (EU), approximately 26% of the overall population smoke [4] and 19.7% do so daily [5]. In particular, in 14 out of the 27 EU Member States, at least one in five citizens is a smoker, with Bulgaria reporting the highest percentage of daily or occasional smokers (34.7% of the population), followed by Greece (32.5%) and Hungary (27.5%) [5]. These three countries alongside Italy, Spain, Poland, Croatia, and France are also the major tobacco producers of the EU, accounting for 99% of its tobacco production [6]. However, tobacco is slowly losing favor within the EU [6].

Ever since the late 1970s, smoking has been recognized as a health hazard [7]. In the decades that followed, numerus studies confirmed that smoking is a leading factor in cardiovascular diseases, strokes, cancer, and many more [8]. According to some studies, the use of tobacco could result in up to 1 billion deaths by the end of the 21st century [3]. Moreover, the cultivation of tobacco and the manufacturing of smoking products have been proven to contribute to climate change as they emit significant amounts of greenhouse gasses (GHGs) [9]. To put that in perspective, the global tobacco industry emits approximately 84 million tons of CO_2_ into the atmosphere every year [9]. This has forced the EU to act and pass legislations that aim to control tobacco use [10]. As a result, during the last two decades, tobacco farms in the EU have been halved and the average annual production has been reduced by nearly 65% [6], yet many criticize the Commission’s tobacco-related policy as ineffective [11].

Despite the EU’s best efforts, the number of smokers (and especially adolescents and young adults) remains high. One of the solutions that has been proposed as a means to mitigate the health hazards of and dependency to tobacco is the use of smoking herbal blends or herbal cigarettes [12]. Herbal cigarettes are popular in Asia, where they have been advertised as healthier and safer alternatives to tobacco, occasionally with beneficial properties for the consumers’ health [13]. In the West, herbal smoking blends are often used in cannabis-substituting herbal mixtures that usually contain synthetic cannabinoids [14]. Nonetheless, the literature mentions non-narcotic herbal blends that could be smoked for recreational purposes.

The aims of the present study were to (i) conduct a systematic review in order to compile the possible tobacco-alternative crops (TACs), (ii) concisely evaluate the potential of incorporating the most prevalent (in the existing literature) TACs in the European tobacco industry, and (iii) assess their utility in smoothening the process of abandoning tobacco, and eventually smoking products altogether, in the EU. The assessment was based on their possible economic and environmental benefits and did not account for the alleged health benefits of the TACs.

## 2. Results

**Figure 1:**
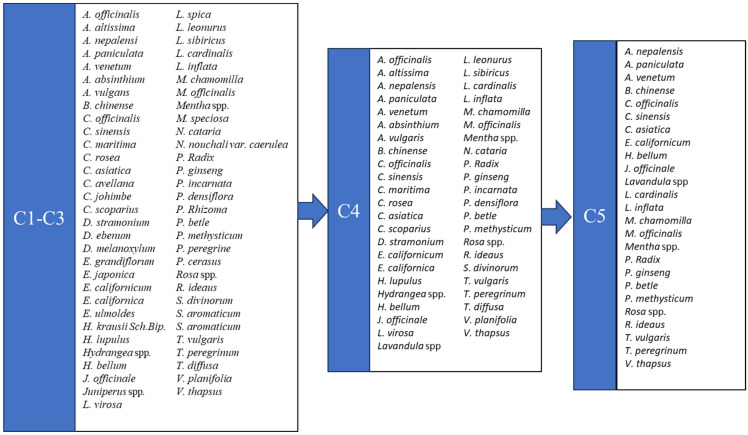
List of TACs through the 2nd stage of the systematic review. C1–C5 represent the exclusion criteria (C1, relevance; C2, use; C3, compliance with EU regulations; C4, classification; C5, toxicity and narcotic substances). Through C1–C3, no species were excluded.

Upon completing the data collection, our reference list included more than 1200 published works. Through the first scan during stage 1, the list was reduced to nearly 900 published documents that mentioned 62 different plant species. Following the exclusion criteria C1–C5 (stage 2), the references list was reduced to 11 [13,15,16,17,18,19,20,21,22,23,24], which mentioned 25 different plant species (Figure 1). Out of these 25 species, 6 were most frequently mentioned in the literature and/or in existing herbal smoking products. In particular, mentha (*Mentha* spp. L.), mullein (*Verbascum thapsus* L.), ginseng (*Panax ginseng* C.A.Mey.), tea (*Camellia sinensis* (L.) Kuntze), calendula (*Calendula officinalis* L.), and chamomile (*Matricaria chamomilla* L.) were the most frequently mentioned species in the literature (Table 1).

## 3. Tobacco Alternative Crops

### 3.1. Calendula

*Calendula officinalis* L., also known as calendula or marigold, is a perennial flowering plant in the Asteraceae family [25]. Originating from the Mediterranean region, calendula has been cultivated for centuries mainly due to its medicinal properties [26], though nowadays it is regarded as a multipurposed crop [27] as it has applications in the pharmaceutical, agrifood, and industrial sectors [27], and it can also be included in herbal mixtures for cigarettes [22].

Calendula acclimatizes to temperate regions [28], but it is susceptible to both frosts and heat stress [26,28]. According to the findings of Eberle et al. [28], the optimum germination temperature is 16–17 °C, and temperatures exceeding 40 °C could result in complete failure of germination. Following crop establishment, the optimum mean temperature for *C. officinalis* has been estimated at 12.5–20.5 °C [26]. The water needs of the crop can vary vastly. In a study by Massoud et al. [29], the authors found that in sandy soils, and in combination with the application of organic fertilizers, the water use efficiency of the crop was optimized at approximately 270 mm/season. The use of organic fertilization in calendula has reported promising results, and according to El-Fatah et al. [30], organic fertilizers can replace 50% of synthetic fertilizers without compromising the performance of the crop. Overall, fertilization, and particularly nitrogen (N) fertilization, is believed to improve the yields and the agronomic characteristics of calendula [31]. The crop’s requirements in N fertilization (as suggested by the literature) ranges from 90–200 kg·N·ha^−1^ and depends on several factors (e.g., the soil properties) [31,32,33,34]. Lastly, calendula exhibits notable potential for breeding salinity- and/or drought-tolerant cultivars [35,36].

### 3.2. Tea

*Camellia sinensis* (L.) Kuntze, commonly referred to as tea, originates from China [37]. This perennial shrub of the Theaceae family is currently being cultivated in more than 100 countries and the beverage it produces is one of the most consumed around the world [38,39]. The popularity of *C. sinensis* partially derives from several pharmaceutical properties that have been attributed to this beverage [40]. In some parts of the world, herbal cigarette brands often include it in their products [19].

According to the literature, the most critical aspects of cultivating tea are the photoperiod, the levels of environmental CO_2_, the temperature, and the availability of water [41]. The photoperiod (12.00–12.15 h) regulates shoot growth and bud development [41]. Notably, in some regions (in latitudes beyond 16° to the north or the south), tea plants often fall into a state of “winter dormancy” where they halt their growth. This phenomenon is instigated in photoperiods of less than 11.15 h and minimum temperatures below 13 °C (for 6 weeks or more) [42]. The optimum temperatures for tea plants range between 18 and 25 °C, and shoot growth decreases in high temperatures that exceed 30 °C [41]. Similarly, draughts and prolonged water stress could reduce yields by a fifth and inhibit the development of the plants [43]. In an effort to estimate the water needs of tea, Cheruiyot et al. [43] concluded that the critical threshold of soil water content for tea is at approximately 20% *v*/*v*. The crop variety, soil properties, and use of fertilization (amongst others) can determine the irrigation regimes in tea [41,43]. Fertilization in particular can increase the water use efficiency of *C. sinensis* plants [44]. Fertilizers, and especially N fertilizers, have been proven to improve the yield and quality of tea [45]. In China, the average application rate of N fertilization is approximately 530 kg·ha^−1^ [46]. It has been suggested that N fertilization benefits tea as increasing N the supply improves the photosynthetic rate of the plants [47]. The same positive correlation has been reported between the atmospheric CO_2_ concentration and the photosynthetic activity, though the increase in the photosynthetic rates in tea plants exposed to elevated CO_2_ levels was temporal [41].

### 3.3. Ginseng

*Panax ginseng* C.A.Mey. is a perennial herb of the of the Araliaceae family [48]. Considered by many as one of the most (if not the most) influential herbs in traditional Chinese medicine, ginseng has been cultivated for centuries in Asia, where it originates from [49]. Even though ginseng is mainly cultivated due to its pharmaceutical properties and nutritional value [50], it is also one of the most commonly used herbs in herbal smoking blends [13].

The literature regarding the optimization of ginseng cultivation and its adaptability in various environments is rather poor. According to Walia et al. [51], the optimum temperature for *P. ginseng* plant growth is 16–28 °C. Mork et al. [52] had previously found that during the early vegetive stages, the ideal air temperature ranges from 10 to 20 °C, and from the flowering stage and afterwards from 21 to 25 °C. Temperatures higher than 30 °C could inhibit plant growth and damage ginseng plants [53]. Overall, it prefers cool-temperate climates as high temperatures and solar radiation can cause scorches and sunburn on its leaves [53]. Ginseng favors well-draining, fertile, acidic soils with pH close to 5 [51,54]. Soil fertility, and specifically the availability of zinc, manganese, iron, and copper, is crucial for its cultivation [55]. Its fertilization needs have not been thoroughly studied; nonetheless, Sun et al. [56] concluded that the application of 500 kg·N, 150 kg phosphorus (P), and 600 kg potassium (K) per ha increases the yield significantly.

### 3.4. Mullein

*Verbascum thapsus* L., commonly referred to as Mullein, belongs to the Scrophulariaceae family [57]. While originating in Europe and Central Asia, it has distributed throughout the world [58,59]. It is a biennial, herbaceous plant which is found in rocky soils, wastelands, fields, anthropogenic regions, abandoned lands, cultivated areas, and roadsides [58,60]. Mullein has been used in traditional medicine as its leaves and flowers reportedly have analgesic properties [61]. Interestingly, cigarettes made from *V. thapsus* have been proposed as a potential remedy for asthma symptoms [62].

Mullein is characterized by ecotypic differentiation and phenotypic plasticity [63]. It is considered as a weed with excellent adaptability to various environments [60]. The fact that it grows in degraded soils such as heavy-metal-contaminated ones [60], or with low fertility [64], is indicative of its acclimatization potential. Nonetheless, dry, sandy soils with good drainage, and with an average pH 6.5–7.8, are ideal for mullein [65,66]. It prefers cool summers with average temperatures below 22 °C in the warmest month and at least 4 months with temperatures over 10 °C [65]. Germination occurs in a wide temperature range (15–40 °C) and is not affected by light [67]. However, according to Semenza et al. [68], seed germination is inhibited in temperatures under 10 °C in the dark. Mean annual precipitations ranging from 500 to 1500 mm are sufficient for its water needs [65]. It should be mentioned, though, that *V. thapsus* is regarded as drought-tolerant, [69] partially due to the trichomes that cover its leaves and increase stomatal resistance [65].

### 3.5. Mentha

Mentha or mint refers to a group of perennial herbs in the Lamiaceae family [70]. It is a genus of cosmopolitan aromatic plants that can be found all across Asia, Europe, Africa, Australia, and South America [71]. Dried or fresh parts of these plants are extensively used in many industries, including in pharmaceuticals, cosmetics, and food commodities [71,72]. Some mints are also frequently used in cigarettes because of the intense menthol flavor and the cooling effect they provide, which covers the bitterness of tobacco [13].

Among the different mentha species, *M. arvensis* L., *M. spicata* L., *M. aquatica* L., *M. canadensis* L., and *M. × piperita* L. are some of the most economically important ones [73]. These species generally prefer loam–sandy loam soil, rich in humus, with an average pH between 6 and 7.5, and good draining ability [73,74]. However, the climatic requirements of mint depend on the species [73]. For instance, *M. arvensis* thrives in tropical and sub-tropical regions, while temperate regions are optimal for *M. × piperita* [73]. Overall, temperatures ranging from 20 to 26 °C are favorable for vegetative growth in most mentha species (Table 2) [73,75]. Similarly, optimal N fertilization rates are estimated at 80–160 kg·N·ha^−1^, depending on the species [73,76,77]. Perhaps the most critical aspect in menta cultivation is irrigation. Mentha requires significant amounts of water and frequent irrigation [73]. The specific amount varies based on the climate, species, and soil and could approach 1000 mm per season [78,79]. Most species are often susceptible to water stress in the summer and waterlogged in the winter (depending on the climate) [73].

### 3.6. Chamomile

*Matricaria chamomilla* L. (chamomile) is an annual, medicinal herbaceous plant that belongs to the Asteraceae family [85]. Chamomile is indigenous to South and East Europe and to parts of Asia, although it has been distributed worldwide [84,85]. *M. chamomilla* is regarded as of major economic importance, and it is considered one of the most popular herbal crops across the world [88]. Its dried flowers are mainly used in making beverages [89], and in the tobacco industry several brands of herbal cigarettes use it as a major cigarette component [22].

*M. chamomilla* is often described as a weed that can adapt in several soil types and climates and it can be found in vacant places, roadsides, and grasslands [90]. However, fertile soils, in combination with warm days and cool nights, are preferable. The ideal temperatures are from 7 to 26 °C (though it can withstand low temperatures as far as −10 °C) and annual precipitation rates from 400 to 1400 mm (Table 2) [84,85]. According to the literature, the optimal temperature for seed germination ranges between 10 and 20 °C [84]. Regarding its fertilization needs, a balanced nutrient soil consistency promotes higher yield. Farmyard and poultry manure or vermicompost at 10 tons per hectare can be applied before sowing. The fertilization needs have been estimated at 50–60 kg·N·ha^−1^, 50 kg·P_2_O_5_·ha^−1^, and 50 kg·K·ha^−1^ [77,83]. Frequent irrigation promotes higher yields, as the crop requires moister soils, especially after sowing [84], yet *M. chamomilla* is rather undemanding and (to a certain extent) tolerates water deficiency [84,91]. Lastly, chamomile is tolerant to soil salinity and alkalinity [91].

## 4. Compliance with EU Strategies

Through the last decade, the EU has been progressively adopting firmer and stricter policies on tobacco, and on smoking products altogether. In 2014, the European Parliament and the Council of the European Union enacted the Tobacco Products Directive (2014/40/EU) [10], enforcing regulations on the use of certain ingredients in smoking products. By banning the products that contain tobacco flavored with fruits, spices, and herbs, the EU intended to control the circulation of cigarettes that attract young people, in accordance with the World Health Organization Framework Convention on Tobacco Control treaty [92]. Nonetheless, herbal cigarettes are not prohibited. However, the labelling of herbal cigarette unit packets must mention that they are not less harmful for human health or more environmentally friendly [10]. Indeed, the available data dictate that herbal cigarettes could be as detrimental for human health as any other conventional tobacco product [93]. Besides some mentions in traditional medical practices [13], there is no proof beyond reasonable doubt that smoking herbal cigarettes could be beneficial, or even less harmful than tobacco. Unless future studies prove otherwise, the potential of adopting TACs can only be measured based on their benefits for the environment, the agricultural sector, and the smoking industry.

The crops that could be considered for replacing (at least partially) tobacco within the EU should also comply with the active agri-environmental policies of the Commission. Case in point, for the aforementioned crops to be successfully incorporated in the European tobacco industry they should align with the European Green Deal, the Farm to Fork strategy, the Common Agricultural Policy 2023–2027, etc. In simple terms, the introduction of TACs should at least promote industrial sustainability, organic farming, biodiversity, a fairer income for farmers and rural development, a reduction in chemical inputs in agriculture, and the minimization of GHG emissions [94,95].

The EU legislation prevents herbal cigarette packages from claiming that they benefit the environment; however, the introduction of alternative crops that can have a positive impact on some agri-environmental aspects has been suggested [96,97]. The literature suggests that the production of 1 kg of dried tobacco requires over 3400 L of water and emits close to 13 kg carbon dioxide equivalent (CO_2eq_) into the atmosphere [98]. In some studies, the carbon footprints of tea and some mint species have been estimated close to 7 kg CO_2eq_/kg dry leaves [99] and 0.02 kg CO_2eq_/kg fresh leaves [100], respectively. Additionally, the water footprints of calendula, spearmint, and tea have been reported in studies at approximately 3000 L of water/kg dry flowers [29], 60 L of water/kg fresh leaves [100], and 1300 L of water/kg of dry leaves [101], respectively. Despite their significantly lower footprints, these values are indicative and not absolute; nonetheless, the findings of these studies are promising. The irrigation and fertilization needs of these crops, and therefore their footprints, can vary depending on the climate and the soil properties. Similarly, the agricultural system and the cultivation practices can significantly increase or decrease the water and carbon footprint of a TAC. In a study by Litskas et al. [100], the carbon footprint of spearmint was halved when grown organically, in comparison to conventional spearmint. Notably, the majority of the aforementioned TACs can perform adequately in organic systems [41,102,103,104,105,106]. This is important as organic farming has also been suggested to promote and benefit biodiversity (both species and ecosystem biodiversity) [107,108]. For instance, in a study by El-Karim et al. [109] regarding arachnid populations, the authors observed that organic chamomile and calendula cultivation resulted in improved biodiversity of arthropods.

Most of the TACs discussed in the present study are multipurpose crops, meaning that they can be used in products besides cigarette manufacturing. This could be beneficial for the income of some producers. Studies have concluded that the multipleness and flexibleness of multipurpose crops offer farmers the opportunity to select where to disseminate their products amongst different markets, based on the most favorable price [110]. For instance, green tea growers could theoretically switch back and forth between the beverage and tobacco industries. The significance of providing farmers with adjustable alternatives becomes evident when considering the market size of tobacco in the EU, and its financial importance. Tobacco is a multibillion-dollar industry with a significant impact on the economy of some Member States. In 2016 alone, tobacco products within the EU generated a total tax revenue that surpassed 100 billion EUR [111]. According to the Tobacco and Nicotine Database of Philip Morris International, in 2021 more than 100,000 people in southern Europe were involved in the tobacco industry (from farmers to retailers) [112]. Amongst them, nearly 70,000 are tobacco farmers. Despite the positive impact that a “smokeless” EU would have on the environment and on the health of its citizens, the process of deserting tobacco presupposes the development of strategies that would consider the employment of the tobacco industry workforce.

## 5. Future Perspectives

**Figure 2:**
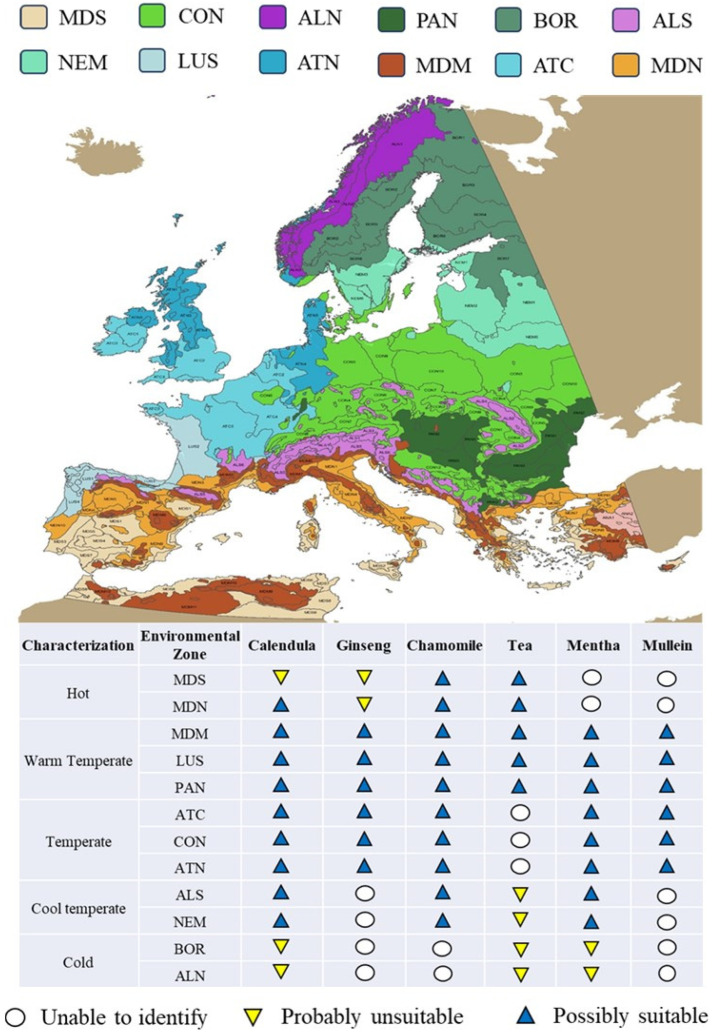
Acclimatization potential of the TACs. The map depicts the environmental stratification of the EU as designed by Metzger et al. [123]. The color legend above the map corresponds to the 12 European environmental zones: MDS, Mediterranean South; MDN, Mediterranean North; MDM, Mediterranean Mountains; LUS, Lusitenean; PAN, Pannonian; ATC, Atlantic Central; CON, Continental; ATN, Atlantic North; ALS, Alpine South; NEM, Nemoral; BOR, Boreal; ALN, Alpine North [123]. The corresponding characterization of the zones is based on the annual temperature sums expressed as growing degree days (with a 0 °C base) [124]. On the table below the map, the blue triangles correspond to the zones that are possibly suitable for each TAC, the yellow triangles to the possibly unsuitable ones, and the white circles to the cases that the authors could not conclude due to limitations in the literature.

The cultivation of the six TACs proposed in the present study is possible in the EU. Calendula, mullein, mentha, and chamomile are indigenous to many parts of Europe [26,59,70,84], and the introduction of tea and ginseng in the EU is supported by studies conducted in France, Spain, and Germany [113,114]. All six species are suitable for warm, temperate, and cool climates [28,73,115,116,117,118,119,120]. Calendula is suitable for cultivation in Mediterranean North regions (according to the Environmental Stratification of Europe) [121], and tea and chamomile can withstand temperatures below 0 °C [84,118]. Figure 2 summarizes the acclimatization potential of the TACs and the possible areas of cultivation, based on the available literature. The propositions of Figure 2 should not be perceived as conclusive. They are estimations based on the environmental requirements of each TAC (soil was not taken into account).

As discussed above, assessing their climate impact, or their potential in reducing the inputs in agriculture and the restoration of biodiversity, is complicated and challenging, yet studies have reported arguably promising results. However, there are major constraints to their incorporation into the European smoking industry. The limited available literature hinders the thorough evaluation of the proposed TACs. The existence of TAC-based smoking products in the Asian markets indicates an advanced technological readiness level for the manufacturing of TAC-based smoking products; however, information regarding the processing or the safety of these products is scarce [13]. From an agronomic point of view, several aspects regarding the optimization of their cultivation require further investigation. On a similar note, the introduction of these crops in new areas presupposes extensive research on their interaction with local ecosystems. For instance, studies argue that mullein should not be cultivated near fisheries or marine ecosystems as its seeds could be toxic to some fish species and damage the normal function of their respiratory system [122]. In theory, these TACs could benefit the tobacco industry and the tobacco growers in the EU, yet the development of a quantifiable indication of their value (e.g., estimations on the % they could improve farmers’ income or reduce the footprint of the tobacco industry) or the development of a TAC framework (on a national and/or EU level) is currently impractical.

Finally, it should be clarified that the opportunities and the weaknesses discussed in the present study regard six crops: calendula, mullein, tea, mentha, chamomile, and ginseng. There are additional plant species and crops that could be considered (Table 1), and even more that did not match the authors’ criteria. Moreover, there are further applications of TACs in the tobacco industry that were not discussed here. Besides cigarette fillings, TACs could be used in heated vapor devices (herbal heat sticks), and the leaves of some species (e.g., *Diospyrus melanoxylon* or *Diospyrus ebemum*) could be used as wrapping materials instead of conventional cigarette rolling papers [125]. Lastly, it is worth mentioning that *N. tabacum* itself has been proposed to have applications in other sectors besides the smoking industry. In fact, the literature suggests that some of its cultivars can be used for biodiesel production [126], as an oil crop feedstock for cosmetics and industrial products [127], and in animal ratios (and particularly in swine nutrition) [128]. These applications could offer farmers new opportunities in a “non-smoking” EU.

Based on the existing policies regarding smoking, it is safe to assume that the Commission aims to reduce the use of tobacco and the consumption of smoking products. Therefore, TACs could benefit the EU tobacco industry provided they conform with the following assumptions:

- They can benefit the income of the actors of the tobacco industry. Whether they can improve farmers’ returns or safeguard the employment of the smoking industry workforce (processors, retailers, etc.) through the transition towards a “smokeless EU”, TACs should be profitable.
- They can be competitive. The acclimatization potential of the discussed TACs does not necessarily translate to suitability for large-scale farming.
- They can reduce the footprint of the industry locally, regionally, or at the EU level. The environmental footprint of a crop could vary vastly amongst different regions. For example, the fertilization and water needs presented in Table 2 are indicative and not absolute. In some regions, tobacco could have a lower footprint than the TACs.
- They are not a permanent alternative feedstock for smoking products. In a future scenario where the workforce of the tobacco industry will be assimilated into other industries, farmers could adopt other industrial, food, or animal feed crops.

## 6. Materials and Methods

### 6.1. Data Collection

Initially, we performed a systematic search on three major scientific databases: Scopus, Web of Science, and PubMed. The key words of this search included “herbal” AND “cigarettes”, “herbal” AND “cigars”, “herbal” AND “smoking” AND “mixtures”, and “smoking” AND “herbal” AND “blend”. We included all document types (articles, reviews, books, conference papers, etc.), regardless of their publication date, and we placed no constraints on which journals were included in the review. The only restriction applied in the literature search was the language selection, as all non-English published works were excluded. Studies that were retrieved in duplicate due to being available in more than one of the databases were filtered, and the repeated ones were removed (stage 1).

### 6.2. Inclusion and Exclusion Criteria

Our first objective was to enlist all plant species that the literature suggests can be included in smoking blends; thus, we scanned the reference list for studies regarding herbal smoking products, and specifically for studies that mentioned the plant species used in these products. These species were sorted out based on the following criteria (C), and any species that did not fulfill them were rejected and not included in our final assessment (stage 2):

1. C1—Relevance. Plant species that can be smoked in cigars and cigarettes. Herbs and herbal mixtures that are used in hookahs, electronic cigars, vaping devices, smokeless tobacco products, etc., were not included.
2. C2—Use. Plant species that can be smoked for recreational and not strictly medical purposes.
3. C3—Compliance with EU regulations. Plant species that cannot be introduced or cultivated in any part of the EU (e.g., invasive alien plant species) were immediately rejected.
4. C4—Classification. We included only herbs, flowering plants, and shrubs. Trees and water lilies were excluded.
5. C5—Toxicity and narcotic substances. Plant species that are mentioned in the literature as narcotics, or that contain substances that could be toxic when smoked, were immediately rejected. We also chose to exclude any plant species that reportedly have psychotropic effects regardless of their legal status.

### 6.3. Data Analysis

Following the exclusion of the studies that did not fulfil C1–C5, the list of TACs for the production of herbal cigarettes included several different plant species. Using descriptive statistics, we attempted to select the most compelling ones. The criterion for this task was their presence in the herbal cigarette industry, expressed as the percentage of relevant manuscripts that mention them and the percentage of existing smoking products (based on the available literature) that include them (stage 3).

## 7. Conclusions

TACs could offer new opportunities in the EU tobacco industry and agricultural sector. However, further research is required. The benefits of TACs are mainly economic in nature, but they could also possibly reduce the climate impact of the tobacco industry. Tea, mullein, ginseng, chamomile, and calendula are suitable candidates for the production of herbal cigarettes in the EU, yet under no circumstances should they be regarded as safer than regular tobacco. Herbal cigarettes or any other herbal-mixture-based smoking products should comply with the objectives of the agri-environmental strategies of the EU, as well as with its legislation that aspires to preserve public welfare.

## Figures and Tables

**Table 1:** Summary of the TACs that were included in studies and smoking products, following the C1–C5 exclusion criteria. The species above the borderline are the ones that were more frequently mentioned. Next to each species are presented the number of studies that mentioned them, their respective percentage of mentions in the literature, the number of smoking products that mentioned them, and their respective percentage of mentions in the product list.

Species	No. of Studies	%	No. of Products	%
*Mentha* spp.	6	12.50	3	10.00
*Verbascum thapsus*	4	8.33	3	10.00
*Panax ginseng*	3	6.25	4	13.33
*Camellia sinensis*	3	6.25	4	13.33
*Matricaria chamomilla*	2	4.17	3	10.00
*Calendula officinalis*	3	6.25	-	-
*Lavandula* spp.	2	4.17	2	6.67
*Eriodictyon californicum*	2	4.17	1	3.33
*Jasminum officinale*	2	4.17	1	3.33
*Apocynum venetum*	2	4.17	1	3.33
*Andrographis paniculata*	2	4.17	1	3.33
*Rosa* spp.	2	4.17	1	3.33
*Paeoniae Radix*	1	2.08	2	6.67
*Tropaeolum peregrinum*	1	2.08	1	3.33
*Bupleurum chinense*	1	2.08	1	3.33
*Melissa officinalis*	2	4.17	-	-
*Rubus ideaus*	2	4.17	-	-
*Anaphalis nepalensis*	1	2.08	-	-
*Centella asiatica*	1	2.08	-	-
*Hypericum bellum*	1	2.08	-	-
*Lobelia cardinalis*	1	2.08	-	-
*Lobelia inflata*	1	2.08	-	-
*Piper betle*	1	2.08	-	-
*Piper methysticum*	1	2.08	-	-
*Thymus vulgaris*	1	2.08	-	-
*Curculigo orchioides*	-	-	1	3.33
*Epimedium grandiflorum*	-	-	1	3.33

**Table 2:** TAC and tobacco needs and requirements. The “Temperature” column refers to the optimal temperatures for plant development; the “Soil” column refers to the preferable soil type and/or properties for crop establishment; the “Fertilization” and “Water needs” columns refer to the annual fertilization (kg·ha^−1^) and water (mm) needs of the crops, respectively; the “General remarks” column includes noteworthy characteristics of each crop; and the “References” column includes the published studies from which the depicted information was obtained. The information presented in the table are indicative and could vary in different areas and agricultural systems.

	Temperature	Soil	Fertilization	Water Needs	General Remarks	References
Calendula	12.5–20.5 °C	Wide range; prefers well drained, pH 6–7	90–200 kg·N·ha^−1^	270 mm per season	Salinity- and drought-tolerant cultivars	[26,29,34,35,36,80]
Mulliein	10–22 °C	Wide range; prefers dry, sandy, good draining, pH 6.7–7.8	-	500–1500 mm per season	Adapts in drought and soils with low fertility	[64,65,69]
Tea	18–25 °C	Requires acidic soil, with pH 4–5.5	530 kg·N·ha^−1^	1200–2200 mm per season	Photoperiods over 11.15 h for 6 weeks or more	[41,42,46,81]
Chamomile	7–26 °C	Wide range, even in soils with low fertility or with pH > 9	50–60 kg·N·ha^−1^, 50 kg P_2_O_5_ ha^−1^, 50 kg·K·ha^−1^	400–1400 mm per season	It tolerates soil alkalinity	[82,83,84,85]
Mentha	20–26 °C	Prefers loam–sandy loam soils, rich in humus, with an average pH between 6 and 7.5	80–160 kg·N·ha^−1^	Could reach 1000 mm per season	Often susceptible to water stress in the summer and waterlogging in the winter	[73,74,76,77,79,81]
Ginseng	16–28 °C	Prefers well-draining, fertile, acidic soils with pH close to 5	500 kg·N·ha^−1^, 150 kg·P·ha^−1^, 600 kg·K·ha^−1^	-	Zinc, manganese, iron, and copper are important for its cultivation	[51,54,55,56]
Tobacco	22–25 °C	Wide range	40–80 kg·N·ha^−1^, 30–90 kg·P·ha^−1^, 50–110 kg·K·ha^−1^	400–600 mm per season	Adopts to a wide range of climates but it is susceptible to frosts	[86,87]

## Data Availability

The data presented in this study are available on request from the corresponding author.
